# Uncomplicated SARS-CoV-2 Infections with Preserved Lung Function in Pediatric Patients with Cystic Fibrosis: A Three-Year Single-Centre Experience

**DOI:** 10.3390/jcm14092979

**Published:** 2025-04-25

**Authors:** Justyna Sieber, Nicole Martin, Klara Schmidthaler, René Gaupmann, Eleonora Dehlink, Alexandra Graf, Zsolt Szépfalusi, Saskia Gruber

**Affiliations:** 1 Division of Pediatric Pulmonology, Allergy and Endocrinology, Department of Pediatrics and Adolescent Medicine, Comprehensive Centre of Pediatrics, Medical University of Vienna, 1090 Vienna, Austria; justyna.sieber@meduniwien.ac.at (J.S.); nicole.martin@meduniwien.ac.at (N.M.); klara.schmidthaler@meduniwien.ac.at (K.S.); rene.gaupmann@meduniwien.ac.at (R.G.); eleonora.dehlink@meduniwien.ac.at (E.D.); zsolt.szepfalusi@meduniwien.ac.at (Z.S.); 2 Institute of Medical Statistics, Centre for Medical Data Science, Medical University of Vienna, 1090 Vienna, Austria; alexandra.graf@meduniwien.ac.at

**Keywords:** cystic fibrosis, COVID-19, SARS-CoV-2, child, susceptibility

## Abstract

**Background/Objectives:** Patients with chronic lung diseases, such as cystic fibrosis, were considered a risk group for a severe course of coronavirus disease 2019 at the beginning of the pandemic. However, mounting evidence suggests that this group may not face an elevated risk for a severe SARS-CoV-2 infection. **Methods**: Here, we present data on the incidence and clinical course of SARS-CoV-2 infections in a single pediatric CF centre in Austria. Clinical variables were analyzed for their potential impact on disease acquisition and severity. A total of 135 young people with CF were assessed from February 2020 until December 2022. **Results**: Eighty-four patients were infected with SARS-CoV-2, out of which nine patients reported re-infection, resulting in 93 SARS-CoV-2 infections. Most infections, 76/93 (82%), occurred during the period of omicron variant predominance. Higher body mass index and respiratory colonization with *Haemophilus influenzae* before the beginning of the pandemic were significantly associated with the risk of acquiring SARS-CoV-2 infection. All patients had an uncomplicated COVID-19 course, regardless of the SARS-CoV-2 variant and COVID-19 vaccine status at infection. The most frequent symptoms were rhinitis (53%), fatigue (49%), cephalea (43%), and fever (38%). Neither oxygen therapy nor hospitalization were needed for any of the patients. Lung function parameters (FEV1, FVC, FEF50, LCI), both in the early post-viral as well as late post-viral stages, were not significantly impacted by SARS-CoV-2 infections. No long-term post-COVID-19 effects were reported. **Conclusions**: Our single-centre experience suggests that the course of SARS-CoV-2 infections in children and adolescents with CF is primarily mild and uncomplicated.

## 1. Introduction

On 5 May 2023, the World Health Organization (WHO) declared the end of COVID-19 as a global public health emergency [1]. However, SARS-CoV-2 infections continue to spread globally, particularly during the winter months, and COVID-19 remains a public health concern, especially for vulnerable populations such as older adults, individuals with underlying health conditions, and the immunocompromised. People with cystic fibrosis (CF) were initially considered to be at a higher risk of severe COVID-19 due to experiences with other respiratory viruses, such as influenza or respiratory syncytial virus (RSV), which often lead to pulmonary exacerbations in this population [2,3,4,5,6,7]. However, later evidence suggested otherwise: the rate of SARS-CoV-2 infection in this group, except for solid organ recipients, was lower than in the general population, and most patients experienced relatively mild courses of the disease [8,9,10,11,12,13,14,15].

This could be caused by the lower median age of the CF population [16] and early infection prevention, involving adherence to mask-wearing, frequent hand hygiene, and self-isolation [17,18]. Other physiological factors that may protect against severe COVID-19 in people with cystic fibrosis (pwCF) have been discussed, including thick mucus secretion in the respiratory tract, pre-existing microbiota, elevated autophagy, and decreased interleukin 6 (IL-6) levels in the respiratory tract [19]. Furthermore, distinct angiotensin-converting enzyme (ACE) expression [20], as well as the impact of medications like dornase alfa [21] or azithromycin [22], have been considered. Additionally, a recent publication has shown lower expression of ribosomal genes in pwCF, which may downregulate the protein translation machinery, thus creating an unfavourable environment for viral replication [14].

Although there is now substantial evidence confirming mild clinical courses of COVID-19 in most pwCF [12,13,14,15,23,24,25], data on risk factors for acquiring the disease or the determinants of different severities of the illness is limited. Even less is known about post-COVID-19 lung function outcomes in this population.

Here, we present a comprehensive analysis of SARS-CoV-2 infections in all pwCF from our pediatric centre in Austria during the first 34 months of the pandemic, including risk factors for acquiring the disease, different clinical courses, as well as lung function results before and after infection. We believe our results contribute to a deeper understanding of the implications of SARS-CoV-2 infections in individuals with cystic fibrosis.

## 2. Materials and Methods

### 2.1. Study Design and Study Population

We systematically assessed all patients treated at our CF centre, starting from the first SARS-CoV-2 infection in Austria in February 2020 with a follow-up until December 2022. Inclusion criteria were age between 0 and 21 years with regular follow-up at our centre, a confirmed diagnosis of cystic fibrosis (CF) based on sweat test and/or genetic testing, and in case of a SARS-CoV-2 infection, available information on testing, either from a certified testing site or based on self-performed antigen tests followed by SARS-CoV-2-specific antibody testing. Informed consent to participate in the study was also required.

Exclusion criteria were age > 21 years, missing or inconclusive data regarding SARS-CoV-2 infection (e.g., positive self-performed antigen test without subsequent SARS-CoV-2 antibody testing), history of solid organ transplantation, or lack of informed consent.

Data collection included details of the clinical presentation, management, and outcome of the SARS-CoV-2 infection, COVID-19 vaccine status, along with patient demographics and potential pre-infection risk factors. These included cystic fibrosis membrane conductance regulator (CFTR) genotype, body mass index (BMI), pre-pandemic lung function (defined as best lung function values in 12 months before the beginning of the pandemic), including forced expiratory volume in one second (FEV1), forced vital capacity (FVC), forced expiratory flow at 50% of vital capacity (FEF50), and lung clearance index (LCI), pancreas insufficiency, CF-related diabetes mellitus (CFRD), allergic bronchopulmonary aspergillosis (ABPA), CFTR modulator therapy, COVID-19 vaccination status, and respiratory microbiology. The most recent lung function and LCI values recorded before SARS-CoV-2 infection were collected, along with three subsequent tests performed after the infection. These results were categorized into pre-infection lung function, early post-viral lung-function (up to 30 days), late post-viral lung-function (up to 90 days), and post-viral recovery lung-function (>90 days).

To confirm SARS-CoV-2 infection, we collected data on positive SARS-CoV-2 PCR tests and positive SARS-CoV-2 antigen self-tests. Additionally, we performed blood sampling for SARS-CoV-2-specific antibodies against nucleocapsid and spike proteins.

The study received ethical approval from the Medical University of Vienna ethics committee (number 1662/2020, approval date: 30 June 2020), and all participants and/or their legal representatives provided written informed consent.

### 2.2. Study Definitions

A confirmed SARS-CoV-2 infection was defined as a history of a positive qPCR test (quantitative real-time polymerase chain reaction) from a legalized testing site. Additionally, positive results of self-performed antigen tests have been acknowledged, but are only considered as confirmation of infection in cases of subsequent positive SARS-CoV-2-specific nucleocapsid antibodies. Patients with positive antigen tests but no subsequent blood sampling for SARS-CoV-2 antibodies were excluded from the analysis.

Symptomatic patients were those who presented COVID-19-like symptoms in the period linked to a confirmed SARS-CoV-2 infection (±5 days).

COVID-19 disease severity was defined as minimal in case of exclusively unspecific symptoms such as fatigue or headache lasting for 1–3 days and without the need for any physician contact; mild in case of short-lasting symptoms (<7 days), mild upper or lower respiratory symptoms, mild gastrointestinal symptoms or short-lasting fever in patients with good general condition, manageable in outpatient settings; moderate in cases of prolonged (>7 days) symptoms with a poor general condition, which could be managed in outpatient settings; and severe in cases of need for hospitalization or oxygen support. The applied COVID-19 severity classification was based on the criteria established by Sieber et al. in their publication on SARS-CoV-2 virus transmission in children [26].

Fully vaccinated at the time of infection was defined as having received at least two doses of a SARS-CoV-2 vaccine prior to the confirmed infection. The majority of patients received the mRNA vaccine Comirnaty^®^ (Pfizer-BioNTech), as this was the first vaccine available, and the only vaccination approved for children in Austria during most of the study period (until October 2022).

### 2.3. Statistical Analysis

Data are presented descriptively using median, 1st quartile (Q1) and 3rd quartile (Q3) (due to non-normally distributed variables and presence of outliers), and sample size (N) as the continuous variables and using numbers and percentages as the categorical variables. Descriptive statistics were calculated separately for patients with or without SARS-CoV-2 infection as well as for all patients.

To evaluate the association between several variables measured before the pandemic (age, BMI, mutation type, sex, pancreatic insufficiency, CFRD, CFTR modulator treatment, FEV1, LCI, airway colonization, glycated hemoglobin (HbA1c)) and the probability of a SARS-CoV-2 infection, two types of analyses were performed. First, univariable generalized linear mixed models accounting for each potential confounding variable as a fixed factor and patient ID as a random effect were calculated. All variables significant in the univariable models were analyzed in a multivariable model. Second, the time to the first infection was analyzed using univariable Cox-proportional hazard regression models. These analyses included only patients already born at the start of the pandemic and accounted only for first infections (*n* = 118).

Furthermore, a possible association between the probability of a more severe SARS-CoV-2 infection and the variables mentioned above at the time of infection was analyzed. A binary endpoint (“asymptomatic/minimal” and “mild/moderate” infection) was evaluated using univariable generalized linear mixed models accounting for each of the potential confounding variables as a fixed factor and patient ID as a random effect. These analyses were calculated for the subgroup of patients with at least one infection and included patients born after the pandemic started.

Pre- and post-SARS-CoV-2 infection lung function parameters (FEV1, FVC, FEF50, LCI) of infected individuals were analyzed with the Wilcoxon matched-pairs signed rank test to evaluate possible post-viral lung function impact.

All *p*-values smaller than 0.05 were considered statistically significant. Due to the exploratory character of the study and the small sample size, no correction for multiplicity was applied.

Analyses were performed using R (version 4.2.2) and SPSS Statistics 29.0. The graphs were created using GraphPad Prism version 9.0.

## 3. Results

### 3.1. Study Population

Data from 135 young pwCF were included in the analyses, out of which 118 born before the onset of the SARS-CoV-2 pandemic were analyzed for possible associations with the risk of acquiring SARS-CoV-2 infection (Table 1). Sex distribution was balanced. Most patients demonstrated a stable lung function with a median best FEV1% of 96.8% pred. (percent predicted) (IR 88.7–107.0) before the onset of the pandemic. Twenty-four patients (20%) were on modulator therapy before the beginning of the pandemic. Among the study patients, seven (6%) suffered from CF-related diabetes mellitus, and three (3%) had an ABPA diagnosis. The median BMI Z score of the study population at the start pf the pandemic was 0.0 (IQR −0.6–0.7). In total, 25 patients (22%) were chronically or intermittently colonized with *Pseudomonas aeruginosa* and 55 (49.1%) with *H. influenzae*, according to Leeds criteria [27].

### 3.2. Incidence of SARS-CoV-2 Infections

**Figure A1:**
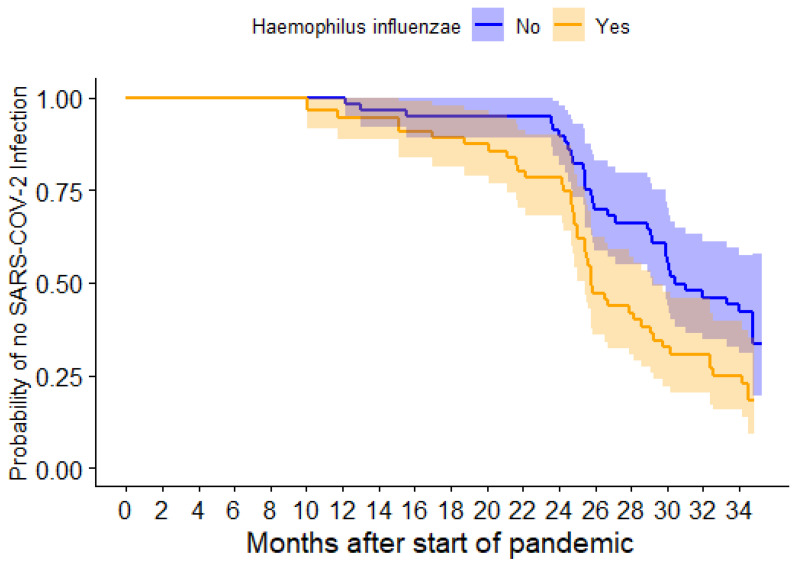
Kaplan–Meier curves for the time to the first SARS-CoV-2 infection and colonization with *Haemophilus influenzae* prior to the onset of the pandemic. Kaplan–Meier curve illustrates an earlier occurrence of a confirmed SARS-CoV-2 infection in patients with a chronic or intermittent *H. influenzae* colonization 12 months before the start of the COVID-19 pandemic (yellow line).

SARS-CoV-2 infection was confirmed in 84 out of 135 analyzed patients (62%). Nine patients tested positive twice, resulting in 93 infections during the study period. Out of 93 SARS-CoV-2 infections, 84 were confirmed by a positive qPCR test and 9 by a positive self-performed antigen test with following positive SARS-CoV-2-specific antibodies against the nucleocapsid antigen (Figure A1).

Based on the date of a confirmed SARS-CoV-2 infection and Austrian epidemiological data (Austrian Agency for Health and Food Safety), each SARS-CoV-2 infection was categorized to the most likely virus variant. The first infection in our study population was reported in December 2020. Most infections, 76/93 (82%), occurred during the time of omicron variant predominance. Specifically, 2/93 (2%) occurred before the emergence of variants of concern, 5/93 (5%) during the time of alpha variant predominance, 1/93 (1%) during the time of beta variant predominance, and 9/93 (10%) during the time of delta variant predominance. The estimated incidence of infection in our study population was 1% for the wild-type virus, 4% for the alpha/beta variants, 7% for the delta variant, and 53% for the omicron variant.

### 3.3. Clinical Presentation of SARS-CoV-2 Infections

Among all infections, 17 cases (18%) were identified as asymptomatic. Twenty-five cases (27%) were categorized as minimal disease severity, forty-six cases (50%) as mild and five cases (5%) as moderate. No severe clinical course was observed. All cases were manageable in an outpatient setting. None of the infected patients needed oxygen support and/or hospitalization. All patients completely recovered, and no long-term post-COVID-19 symptoms have been reported. Uncomplicated disease courses were observed irrespective of SARS-CoV-2 variants, modulator therapy, pancreas sufficiency status, BMI, and airway bacterial colonization. The most frequently reported symptoms were rhinitis (53%), fatigue (49%), cephalea (43%), fever (38%), and increased cough (37%). The median duration of symptoms was three days, ranging from one to fourteen days (IQR 7–1) (Table 2).

The clinical courses of vaccinated and unvaccinated individuals were uncomplicated, and no statistically significant difference in the severity of the disease was seen between both groups. Symptoms did not differ significantly, except for pharyngitis, which was more common in the vaccinated group (*p* = 0.019). Vaccinated patients were older (*p* < 0.001) and less frequently infected with variants other than omicron (*p* < 0.001) (Table A1).

### 3.4. Pre- and Post-SARS-CoV-2 Infection Lung Function

**Figure 1:**
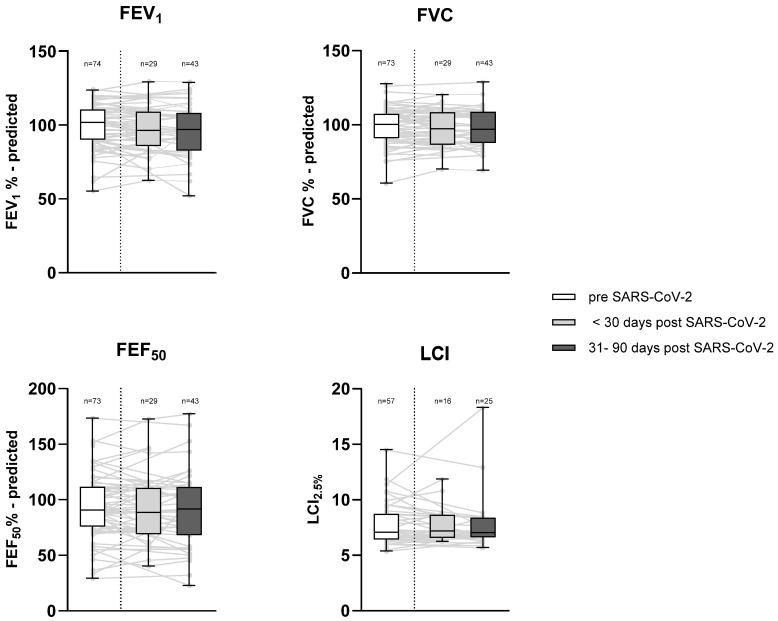
Comparison of lung function pre- and post-SARS-CoV-2 infection. Lung function parameters measured before and after a SARS-CoV-2 infection. Re-infections are included. The dotted line divides the pre- and post-SARS-CoV-2 infection periods. The last lung function tests and LCI measurements obtained before the infection (white) were compared with post-infection measures categorized into the early post-viral stage (up to 30 days, light grey) and late post-viral stage (up to 90 days, dark grey). No significant changes in the above-mentioned lung function parameters were found between the different time points. *n*: number of values obtained for different time points, FEF_50_ = forced expiratory flow at 50%. FVC% = forced vital capacity, FEV_1_% = predicted forced expiratory volume in 1 s (Global Lung Function Initiative Network references [28]), LCI_2.5%_ = lung clearance index.

To assess the potential pulmonary consequences of SARS-CoV-2 infection, we analyzed lung function tests, including FEV1, FVC, FEF50, and LCI values, both before and after SARS-CoV-2 infection, as described in the methods. This analysis was conducted for patients who could undergo lung function testing (*n* = 74). No statistically significant changes in lung function parameters were observed between pre-infection lung function and the early post-viral stage (up to 30 days after infection) or the late post-viral stage (up to 90 days after infection) lung function. Similarly, no statistically significant changes were found between the early and late post-viral lung function. Furthermore, lung function tests performed during the recovery phase (minimum of 90 days after infection) showed no differences from pre-infection measurements or any other post-infection measurements (Figure 1).

### 3.5. Risk of Acquiring a SARS-CoV-2 Infection

To evaluate potential associations between patients’ characteristics and the risk of acquiring SARS-CoV-2 infection, we analyzed all patients who were born before the beginning of the pandemic for the binary outcome, ‘confirmed SARS-CoV-2 infection’ (*n* = 118). Patients with higher BMI and colonized with *H. influenzae* in the last 12 months before the beginning of the pandemic had a significantly higher probability of acquiring a SARS-CoV-2 infection in both uni- and multivariable analysis (*p* = 0.018 and *p* = 0.025 for BMI, *p* = 0.037 and *p* = 0.035 for *H. influenzae*, respectively) (Table 3).

### 3.6. Time to First SARS-CoV-2 Infection

In the univariable Cox regression analysis of ‘time to first SARS-CoV-2 infection’, a significant association between BMI and airway colonization with *H. influenzae* was observed. Higher BMI and *H. influenzae* airway colonization accounted for an earlier infection (*p* = 0.014 and *p* = 0.008). The estimated median time to a confirmed SARS-CoV-2 infection was 25.8 months (95%CL: 25.5–29.3) for patients with pre-pandemic *H. influenzae* colonization and 30.4 months (95%CL: 29.2–34.7) for patients without (Table A2, Figure A1).

### 3.7. Associations with Disease Severity

A significant association between age at infection and the probability of a more severe course of COVID-19 was observed, with older patients showing a larger likelihood for mild or moderate versus none or minimal symptoms (*p* = 0.04). All other variables did not show significant association (Table 4).

## 4. Discussion

We reported the incidence, clinical course, and post-viral lung-function outcomes, as well as potential risk factors, of SARS-CoV-2 infection in young pwCF over 34 months of the COVID-19 pandemic in a European pediatric CF centre. All cases were uncomplicated, regardless of virus variant, vaccination status, or other clinical characteristics. None of the COVID-19-affected CF patients suffered from long-term sequelae.

Conflicting evidence exists concerning both incidence and disease severity of COVID-19 in pwCF. While some studies reported lower COVID-19 incidence compared to the general population [9,11,29], data suggest higher incidence than in the age-adjusted general population [30]. When interpreting these findings, different age distributions in pwCF, as well as higher vigilance, must be considered. While early SARS-CoV-2 testing was established as a standard of care in many CF populations, it was not routinely conducted in many countries in the general population. Likewise, the severity of COVID-19 in pwCF at the beginning of pandemic seemed to be overestimated. Current reports suggest a milder-than-expected course of the disease [12,14,15,24,25]. Likewise, initially, more frequently reported hospital admissions for pwCF [30,31] might be attributable to precautionary measures rather than increased disease severity, which may be supported by a lower case fatality rate in pwCF compared to the general population [30]. The possible selection bias of registry studies due to voluntary reporting of cases and, consequently, over-reporting of more severe ones needs to be considered when evaluating disease severity. Finally, many studies analyze lung-transplanted pwCF together with non-transplant ones [9,10,11,30,32], which may significantly overestimate the incidence and disease severity of COVID-19 in non-transplanted pwCF.

In our single-centre study, children and adolescents with CF were systematically assessed for SARS-CoV-2 clinical symptoms, as well as undergoing SARS-CoV-2 testing, over 34 months of the pandemic. Additionally, routine SARS-CoV-2 testing in Austria was mandatory before each clinic visit and a standard procedure in all school and nursery settings during the SARS-CoV-2 pandemic, thus limiting the rate of undetected asymptomatic infections in our cohort.

The first reported SARS-CoV-2 infection in our study population occurred in December 2020, and the infection rate increased steadily over the following months. The cumulative incidence of particular virus variants varies from 1% for wild-type infection to 53% for the omicron variant. Overall, uncomplicated disease courses were observed irrespective of the SARS-CoV-2 variants.

The clinical symptoms of COVID-19 in our study population were similar to those reported for the non-CF populations, with fever, fatigue, and rhinitis being most commonly reported.

Other studies reported on older age [8,10,33], post-transplant status [10,11,30,32,33], CF-related diabetes [10,33], and lower lung function [10,33] as risk factors for more severe COVID-19 and/or higher incidence of SARS-CoV-2 infection in pwCF. Our analysis confirmed that age may be associated with disease severity, with older patients presenting more symptoms. No other variable showed significant results regarding disease severity. However, since none of the analyzed SARS-CoV-2 infections were severe, this observation must be interpreted cautiously.

The uncomplicated courses of COVID-19 in our study group are consistent with recent reports suggesting better-than-expected outcomes of COVID-19 in the CF population [10,11,12,14,24,25,32,34] and good outcomes in the pediatric population in general [35,36].

The low median age, stable CF disease with well-preserved lung function and low rates of CF-related complications in our cohort might contribute to these favourable results.

While lung function decline in pwCF has been previously associated with viral infections with H1N1 [6], respiratory syncytial virus (RSV) [37], and influenza B [38], we did not observe any significant changes in lung function or LCI after a SARS-CoV-2 infection. This observation contrasts with evidence on post-COVID-19 lung function changes in the non-CF population, such as altered diffusion capacity of the lungs, restrictive and/or obstructive patterns [39,40,41], although these results were mainly obtained in patients after severe COVID-19 courses. Data on post-COVID-19 lung function changes in pwCF are scarce [23,24,29]. Thus, our results of preserved early post-viral, as well as late post-viral, lung function measurements contribute significantly to the knowledge about pulmonary function following SARS-CoV-2 infection in pwCF.

We observed an association between BMI and susceptibility to SARS-CoV-2 infection. While a higher BMI has previously been reported as a risk factor for higher susceptibility to COVID-19 in the general population [42,43], it is noteworthy that this association could also be demonstrated in our patient collective, considering the lower median BMI in pwCF and only a few obese (>95th percentile), and there being no morbidly obese (>99th percentile) patients in our cohort. As opposed to the general population [44,45], we did not find any association between BMI and COVID-19 disease severity.

Interestingly, we also found an association between susceptibility to SARS-CoV-2 infection and *H. influenzae* colonization. To our knowledge, no data have been published on *H. influenzae* as a risk factor for SARS-CoV-2 acquisition. While it has been suggested that colonization with *H. influenzae* may be associated with the fibrotic phenotype of COVID-19, and consequently with idiopathic pulmonary fibrosis [46], we did not find more severe courses of COVID-19 in patients colonized with *H. influenzae* or a negative effect on subsequent lung function. In pwCF, *H. influenzae* has been demonstrated to be associated with chronic airway inflammation [47], which may influence viral–bacterial interactions and predispose individuals to viral infections. The incidence of *H. influenzae* colonization in pwCF is highest among preschool and early-school-age children [48], which may indicate greater community exposure, and thus represent another possible explanation for the higher incidence of SARS-CoV-2 in colonized individuals.

Several patients started with a highly effective modulator therapy during the SARS-CoV-2 pandemic, which could have potentially changed the susceptibility to viral infection, its course, and outcomes [49,50,51]. This assumption could not be confirmed in our analysis, patients under modulator therapy did not show lower susceptibility or milder courses of the disease.

The COVID-19 vaccine [52] was introduced gradually for the pediatric population in Austria, beginning in May 2021. Among 93 confirmed SARS-CoV-2 infections, 44 occurred in COVID-19-unvaccinated children. We did not observe any significant difference in the severity of COVID-19 between vaccinated and unvaccinated patients. Pharyngitis was more frequently reported in the vaccinated group, which may be both a variant-specific and/or age-specific observation. The difference in age and infecting variant between vaccinated and unvaccinated patients can be explained by the stepwise introduction of vaccination in the pediatric population, resulting in vaccinated patients being older and mostly infected with the omicron variant.

Despite the systematic study approach, our cohort represents a relatively small sample size, resulting in limited statistical power. To mitigate any possible recall or reporting bias, every patient was interviewed in a detailed and standardized manner, allowing for a comprehensive analysis of the SARS-CoV-2 clinical course, potential risk factors, and long-term sequelae. Additionally, population-wide monitoring through easily accessible, free testing and regular routine testing in all public institutions in Austria helped reduce the false-negative rate in our cohort [53].

Our study describes uncomplicated clinical courses of COVID-19, based on the assessment of clinical symptoms. Due to the asymptomatic or mild nature of the disease in our study population and the young age of the patients, the management of COVID-19 was limited to clinical advice only. Further invasive diagnostics, such as blood sampling, were not routinely performed during the acute phase of the disease. Consequently, we do not have data on laboratory markers previously described as predictors of disease severity, such as the neutrophil-to-lymphocyte ratio [54,55]. Thus, it is unlikely that these assessments would have influenced our clinical management in this exclusively mildly affected population.

## 5. Conclusions

The courses of SARS-CoV-2 infection in the pediatric CF population may be more favourable than initially feared. Children suffered a mild course of disease independently of the SARS-CoV-2 variant, existing comorbidities, or vaccination status. Higher BMI and colonization with *H. influenzae* seem to be associated with the risk of acquiring SARS-CoV-2, while older age at infection seems to induce slightly more symptoms. Our data may have further implications on SARS-CoV-2 preventive measures and clinical management for COVID-19 in pwCF.

## Figures and Tables

**Table 1:** Characteristics of pediatric people with CF born before the start of the pandemic.

		Total	Not Infected *	Infected *
Overall	*n*	118	39	79
Sex	Female, *n* (%)	53 (44.9)	21 (39.6)	32 (60.4)
	Male, *n* (%)	65 (55.1)	18 (27.7)	47 (72.3)
Age (years)	Median (IQR)	9.7 (4.7–12.7)	9.3 (3.6–12.6)	9.7 (5.21–13.09)
Age group	<5, *n* (%)	31 (26.3)	11 (35.5)	20 (64.5)
	5–10, *n* (%)	30 (25.4)	9 (30)	21 (70)
	>10–15, *n* (%)	42 (35.6)	13 (31)	29 (69.1)
	>15, *n* (%)	15 (12.7)	6 (40)	9 (60)
BMI Z score ⊥	Median (IQR)	−0.01 (−0.16–0.68)	−0.34 (−1.14–0.35)	0.1 (−0.38–0.79)
BMI group ^1^	Underweight, *n* (%)	3 (2.6)	2 (66.7)	1 (33.3)
	Normal weight, *n* (%)	97 (82.2)	31 (32.6)	66 (68.7)
	Overweight, *n* (%)	14 (12.1)	4 (28.6)	10 (71.4)
	Obesity, *n* (%)	3 (2.6)	1 (33.3)	2 (66.7)
CFTR mutation type	dF508 homozygous, *n* (%)	45 (38.1)	14 (31.1)	31 (68.9)
	dF508 heterozygous, *n* (%)	48 (40.7)	17 (35.4)	31 (64.6)
	other, *n* (%)	25 (21.2)	8 (32)	17 (68)
Pancreatic insufficiency	No, *n* (%)	21 (17.5)	6 (28.6)	15 (71.4)
	Yes, *n* (%)	97 (82.5)	33 (34.0)	64 (66.0)
CFRD	No, *n* (%)	110 (94.0)	36 (32.7)	74 (67.3)
	Yes, *n* (%)	7 (6.0)	2 (28.6)	5 (71.4)
Lowest HbA1c ⊥	Median (IQR)	5.5 (5.3–5.7)	5.6 (5.3–5.7)	5.4 (5.2–5.7)
ABPA	No, *n* (%)	114 (97.4)	37 (32.5)	77 (67.5)
	Yes, *n* (%)	3 (2.6)	1 (33.3)	2 (66.7)
**CFTR modulator therapy**				
Modulator	No, *n* (%)	94 (79.7)	32 (34.0)	62 (66)
	Yes, *n* (%)	24 (20.3)	7 (29.2)	17 (70.8)
Modulator type	HEMT, *n* (%)	4 (3.4)	1 (25)	3 (75)
	No HEMT, *n* (%)	20 (17.0)	6 (30)	14 (70)
**Lung function ⊥**				
FEV1% pred.	Median (IQR)	96.8 (88.7–107.0)	96.4 (90.0–104.5)	96.8 (88.5–109.8)
FEV1 Z score	Median (IQR)	−0.27 (−0.96–0.61)	−0.31 (−0.83–0.37)	−0.27 (−0.96–0.8)
FEV1 best ‡	<70, *n* (%)	4 (4.7)	2 (50)	2 (50)
	≥70, *n* (%)	82 (95.4)	24 (29.3)	58 (70.7)
Lowest LCI	Median (IQR)	7.7 (7.0–9.1)	7.79 (7.06–9.11)	7.74 (6.79–9.04)
**Airway colonization ^2^**				
*Pseudomonas aeruginosa*	No, *n* (%)	87 (77.7)	27 (31.0)	60 (69)
	Yes, *n* (%)	25 (22.3)	9 (36)	16 (64)
*Achromobacter xylosoxidans*	No, *n* (%)	105 (93.8)	34 (32.4)	71 (67.6)
	Yes, *n* (%)	7 (6.3)	2 (28.6)	5 (71.4)
*Serratia marcescens*	No, *n* (%)	109 (97.3)	34 (31.2)	75 (68.8)
	Yes, *n* (%)	3 (2.7)	2 (66.7)	1 (33.3)
*Burkholderia cepacia*	No, *n* (%)	110 (98.2)	35 (31.8)	75 (68.2)
	Yes, *n* (%)	2 (1.8)	1 (50)	1 (50)
*Aspergillus fumigatus*	No, *n* (%)	99 (88.4)	30 (30.3)	69 (69.7)
	Yes, *n* (%)	13 (11.6)	6 (46.2)	7 (53.9)
*Staphylococcus aureus*	No, *n* (%)	11 (9.8)	2 (18.2)	9 (81.8)
	Yes, *n* (%)	101 (90.2)	34 (33.7)	67 (66.3)
*Haemophilus influenzae*	No, *n* (%)	57 (50.9)	24 (42.1)	33 (57.9)
	Yes, *n* (%)	55 (49.1)	12 (21.8)	43 (78.2)
*Mycobacterium nontuberc.*	No, *n* (%)	111 (99.1)	36 (32.4)	75 (67.6)
	Yes, *n* (%)	1 (0.9)	0 (0%)	1 (100)
Fully COVID-19-vaccinated	No, *n* (%)	40 (33.9)	7 (17.5)	33 (82.5)
	Yes, *n* (%)	78 (66.1)	32 (41)	46 (60)

* A confirmed SARS-CoV-2 infection was defined as a history of a positive qPCR test from a legalized testing site or positive results of self-performed antigen tests with subsequent positive SARS-CoV-2 specific nucleocapsid antibodies. Patients with positive antigen tests but no subsequent blood sampling for SARS-CoV-2 antibodies were excluded from the analysis. ‡ = Best FEV1% measured within 12 months before the start of the pandemic (below and above 70% FEV1% predicted). ⊥ pre-pandemic best value between 31 January 2019, and 1 February 2020. ^1^ *n* = 116, two patients switched care to our clinic after the start of the pandemic, BMI data before pandemic are missing. ^2^ *n* = 112, two patients switched care to our clinic after the start of the pandemic, airway colonization data are missing; two patients were diagnosed shortly before start of pandemic and no swab tests are available, the swabs of two patients could not be classified according to Leeds criteria. ABPA = allergic bronchopulmonary aspergillosis, BMI = body mass index, CF = cystic fibrosis, CFRD = CF-related diabetes, CFTR = cystic fibrosis transmembrane conductance regulator, CI = confidence interval, CL = confidence level, FEV1 = predicted forced expiratory volume in 1 s (Global Lung Function Initiative Network references), HbA1c = glycated hemoglobin, CFTR= cystic fibrosis transmembrane conductance regulator, HR = hazard ratio, LCI = lung clearance index, *Mycobacterium nontuberc.* = nontuberculous mycobacteria, OR = odds ratio. Airway colonization according to Leeds criteria 12 months before beginning of pandemic (1 February 2020).

**Table 2:** Clinical presentation of SARS-CoV-2 infections in confirmed cases (including nine re-infections).

		SARS-CoV-2 Variants			
Variable	Total	Wild Type	Alpha/Beta	Delta	Omicron
Overall, *n*	93	2	6	9	76
Age at infection (years), median (IQR)	11.3 (6.1–15.1)	16.2 (14.9–17.5)	6.5 (5.4–11.8)	7.9 (5.8–10.1)	11.7 (6.5–15.2)
Female, *n* (%)	39 (41.9)	1 (50.0)	3 (50.0)	5 (55.6)	30 (39.5)
Male, *n* (%)	54 (58.1)	1 (50.0)	3 (50.0)	4 (44.4)	46 (60.5)
**Disease severity**					
Asymptomatic, *n* (%)	17 (18.3)	1 (50.0)	1 (16.7)	1 (11.1)	14 (18.4)
Minimal, *n* (%)	25 (26.9)	0 (0)	1 (16.7)	4 (44.4)	20 (26.3)
Mild, *n* (%)	46 (49.5)	1 (50.0)	2 (33.3)	4 (44.4)	39 (51.3)
Moderate, *n* (%)	5 (5.4)	0 (0.0)	2 (33.3)	0 (0.0)	3 (3.9)
Severe, *n* (%)	0 (0.0)	0 (0.0)	0 (0.0)	0 (0.0)	0 (0.0)
**COVID-19-like symptoms**					
Duration (days), median (IQR)	3 (1–7)	2.5 (1.2–3.8)	7 (5.5–8.5)	2 (1.8–4.8)	3 (1–7)
Fever, *n* (%)	35 (38.0)	1 (50.0)	2 (33.3)	2 (22.2)	30 (40)
Fatigue, *n* (%)	45 (48.9)	1 (50.0)	1 (16.7)	3 (33.3)	40 (53.3)
Increased cough, *n* (%)	34 (37.0)	1 (50.0)	4 (66.7)	3 (33.3)	26 (34.7)
Pharyngitis, *n* (%)	24 (26.4)	1 (50.0)	1 (16.7)	2 (25.0)	20 (26.7)
Dyspnea, *n* (%)	5 (5.4)	1 (50.0)	2 (33.3)	0 (0.0)	2 (2.7)
Chest tightness, *n* (%)	3 (3.3)	1 (50.0)	1 (16.7)	0 (0.0)	1 (1.3)
Wheezing, *n* (%)	4 (4.3)	1 (50.0)	2 (33.3)	0 (0.0)	1 (1.3)
Increased sputum, *n* (%)	6 (6.5)	0 (0.0)	1 (16.7)	1 (11.1)	4 (5.3)
Hemoptysis, *n* (%)	1 ** (1.1)	0 (0.0)	0 (0.0)	0 (0.0)	1 ** (1.3)
Rhinitis, *n* (%)	48 (52.7)	1 (50.0)	3 (50)	5 (62.5)	39 (52)
Conjunctivitis, *n* (%)	4 (4.3)	0 (0.0)	2 (33.3)	0 (0.0)	2 (2.7)
Anosmia, *n* (%)	6 (6.5)	1 (50.0)	2 (33.3)	1 (11.1)	2 (2.7)
Ageusia, *n* (%)	7 (7.6)	1 (50.0)	2 (33.3)	0 (0.0)	4 (5.3)
Diarrhea, *n* (%)	3 (3.3)	1 (50.0)	0 (0.0)	0 (0.0)	2 (2.7)
Myalgia/Arthralgia, *n* (%)	13 (14.3)	1 (50.0)	2 (40)	1 (11.1)	9 (12.0)
Cephalea, *n* (%)	39 (42.9)	1 (50.0)	3 (50)	2 (25.0)	33 (44.0)
Vomiting, *n* (%)	7 (7.6)	0 (0.0)	1 (16.7)	0 (0.0)	6 (8.0)
Abdominalgia, *n* (%)	7 (7.6)	0 (0.0)	1 (16.7)	0 (0.0)	6 (8.0)
**COVID-19 vaccination status at time of infection**					
Fully vaccinated, *n* (%)	49 (52.7)	0 (0.0) *	0 (0.0) *	1 (11.1)	48 (63.2)
Unvaccinated, *n* (%)	44 (47.3)	2 (100.0) *	6 (100.0) *	8 (88.9)	28 (36.8)

Clinical presentation of SARS-CoV-2 infections in confirmed cases (including nine re-infections). IQR = interquartile range * = COVID-19 vaccine not available at that time, ** pre-existing hemoptysis.

**Table 3:** Associations with the risk for a SARS-CoV-2 infection in young people with CF.

		Univariable		Multivariable	
Variable	Comparison	OR (95% CI)	*p*-Value	OR (95% CI)	*p*-Value
Age (years)		1.024 (0.940–1.116)	0.588	1.043 (0.949–1.147)	0.379
BMI Z scores		1.902 (1.119–3.234)	0.018	2.009 (1.093–3.692)	0.025
CF mutation type	dF508 heterozygous vs. dF508 homozygous	0.848 (0.326–2.206)	0.736		
	other vs. dF508 homozygous	0.924 (0.289–2.951)	0.894		
Sex	Male vs. female	1.884 (0.776–4.574)	0.161		
Pancreatic insufficiency	Yes vs. no	0.852 (0.267–2.719)	0.787		
CFRD	Yes vs. no	1.342 (0.215–8.369)	0.753		
CFTR modulator	Yes vs. no	1.293 (0.441–3.784)	0.640		
CFTR modulator type	HEMT vs. no	1.472 (0.116–18.613)	0.765		
	No HEMT vs. no	1.263 (0.401–3.975)	0.690		
FEV1% *		1.014 (0.978–1.051)	0.460		
FEV1 Z score *		1.193 (0.770–1.848)	0.430		
Lowest HbA1c *		1.011 (0.365–2.803)	0.983		
Lowest LCI *		0.931 (0.703–1.234)	0.619		
*Pseudomonas aeruginosa*	Yes vs. no	0.796 (0.284–2.230)	0.664		
*Achromobacter xylosoxidans*	Yes vs. no	1.328 (0.215–8.187)	0.760		
*Serratia marcescens*	Yes vs. no	0.169 (0.010–2.883)	0.219		
*Burkholderia cepacia*	Yes vs. no	0.385 (0.016–9.191)	0.555		
*Aspergillus fumigatus*	Yes vs. no	0.423 (0.110–1.626)	0.210		
*Staphylococcus aureus*	Yes vs. no	0.403 (0.072–2.249)	0.300		
*Haemophilus influenzae*	Yes vs. no	2.987 (1.069–8.347)	0.037	3.363 (1.091949–10.367)	0.035

The risk of SARS-CoV-2 infection in patients with CF and the statistically significant results. Results of the logistic regression models for infection were calculated with a binary endpoint (yes/no), odds ratios (OR), and corresponding confidence intervals, as well as *p*-values. * Pre-pandemic best value between 31 January 2019 and 1 February 2020. BMI = body mass index, CF = cystic fibrosis, CFTR = cystic fibrosis transmembrane conductance regulator, CI = confidence interval, FEV1 = predicted forced expiratory volume in 1 s, HEMT = highly effective modulator treatment, LCI = lung clearance index, OR = odds ratio, *P. aeruginosa* = *Pseudomonas aeruginosa*. Airway colonization according to Leeds criteria 12 months before beginning of pandemic (=1 February 2020).

**Table 4:** Risk of a more severe (mild/moderate) clinical course of a SARS-CoV-2 infection for young people with CF.

Variable	Comparison	OR (95% CI)	*p*-Value
Age at infection		1.097 (1.004–1.199)	**0.040**
BMI Z scores		1.132 (0.975–1.313)	0.104
CFTR mutation type	F508del heterozygous vs. F508del homozygous	0.937 (0.366–2.399)	0.892
	F508del homozygous vs. other	0.753 (0.234–2.424)	0.635
Sex	Male vs. female	1.071 (0.458–2.503)	0.874
COVID-19-vaccinated	Yes vs. no	2.103 (0.871–5.081)	0.099
Pancreatic insufficiency	Yes vs. no	0.846 (0.264–2.705)	0.778
CFRD	Yes vs. no	1.789 (0.278–11.535)	0.540
CFTR modulator	Yes vs. no	0.858 (0.366–2.012)	0.725
CFTR modulator type	HEMT vs. no	1.065 (0.401–2.831)	0.900
	No HEMT vs. no	0.960 (0.222–4.154)	0.956
FEV1% *		1.023 (0.989–1.058)	0.195
FEV1 Z score *		1.303 (0.868–1.955)	0.202
HbA1c *		1.283 (0.677–2.430)	0.445
LCI *		1.507 (0.826–2.750)	0.181
*Pseudomonas aeruginosa*	Yes vs. no	1.153 (0.376–3.534)	0.804
*Achromobacter xylosoxidans*	Yes vs. no	0.556 (0.104–2.975)	0.493
*Serratia marcescens*	Yes vs. no	0.217 (0.016–2.870)	0.246
*Burkholderia cepacia*	Yes vs. no	0.790 (0.038–16.218)	0.879
*Aspergillus fumigatus*	Yes vs. no	0.363 (0.026–5.077)	0.451
*Staphylococcus aureus*	Yes vs. no	1.806 (0.330–9.900)	0.496
*Haemophilus influenzae*	Yes vs. no	1.099 (0.423–2.860)	0.846
SARS-CoV-2 type	Omicron vs. other	1.095 (0.372–3.223)	0.870
Days after last COVID-19 vaccination		1.001 (0.993–1.009)	0.747

Results of the univariable logistic regression models for infection were calculated with a binary endpoint (yes/no), odds ratios (OR) and corresponding confidence intervals as well as *p*-values. Note that because only one variable showed a significant result in the univariable analyses, no multivariable analysis was performed. BMI = body mass index, CF = cystic fibrosis, CFRD = CF-related diabetes, CFTR = cystic fibrosis transmembrane conductance regulator, CI = confidence interval, FEV1 = predicted forced expiratory volume in 1 s [28], HEMT = highly effective modulator treatment, LCI = lung clearance index, OR = odds ratio, airway colonization according to Leeds criteria 12 months before beginning of pandemic (before 1 February 2020). * Pre-pandemic best value between 31 January 2019 and 1 February 2020.

## Data Availability

The anonymized data presented in this study are available upon request from the corresponding author, due to patient data protection regulations.

## Abbreviations

The following abbreviations are used in this manuscript:

ABPA	Allergic bronchopulmonary aspergillosis
ACE	Angiotensin-converting enzyme
BMI	Body mass index
CF	Cystic fibrosis
CFTR	Cystic fibrosis transmembrane conductance regulator
CFRD	CF-related diabetes
IL-6	Interleukin 6
pwCF	People with cystic fibrosis
SARS-CoV-2	Severe acute respiratory syndrome coronavirus type 2
COVID-19	Coronavirus Disease 2019
qPCR	Quantitative real-time polymerase chain reaction
H1N1	Hemagglutinin type1 and neuraminidase type1
FEV1	Forced expiratory volume in one second
FVC	Forced vital capacity
FEF50	Forced expiratory flow at 50% of vital capacity
LCI	Lung clearance index
ECFSPR	European Cystic Fibrosis Society Patient Registry
% pred.	Percent predicted

## Appendix A

**Table A1:** Clinical course of COVID-19 with respect to vaccine status (only first infections considered).

Variable	Total	Not Vaccinated	Vaccinated	*p*-Value
Overall	84	16	68	
Age at infection (years), median (IQR)	11.6 (6–15.2)	7.4 (3.8–11.9)	14 (9.9–16.8)	**<0.001 *****
Female, *n* (%)	36 (42.9)	19 (50)	17 (37)	0.327
Male, *n* (%)	48 (57.1)	19 (50)	29 (63)	
**COVID-19 like symptoms**				
Duration (days), median (IQR)	4 (1.8–7)	3.5 (2–6.5)	4 (1–7)	0.758
Fever, *n* (%)	34 (40.5)	19 (50)	15 (32.6)	0.164
Fatigue, *n* (%)	43 (51.2)	19 (50)	24 (52.2)	1
Increased cough, *n* (%)	33 (39.3)	18 (47.4)	15 (32.6)	0.248
Pharyngitis, *n* (%)	23 (27.7)	5 (13.5)	18 (39.1)	**0.019**
Dyspnea, *n* (%)	5 (6)	3 (7.9)	2 (4.3)	0.825
Chest tightness, *n* (%)	3 (3.6)	2 (5.3)	1 (2.2)	0.866
Wheezing, *n* (%)	4 (4.8)	4 (10.5)	0 (0)	0.082
Increased sputum, *n* (%)	6 (7.1)	3 (7.9)	3 (6.5)	1
Hemoptysis, *n* (%)	1 * (1.2)	0 (0)	1 * (1.5)	1
Rhinitis, *n* (%)	44 (53)	8 (53.3)	36 (52.9)	1
Conjunctivitis, *n* (%)	4 (4.8)	2 (12.5)	2 (2.9)	0.336
Anosmia, *n* (%)	6 (7.1)	4 (10.5)	2 (4.3)	0.504
Ageusia, *n* (%)	7 (8.3)	3 (7.9)	4 (8.7)	1
Diarrhea, *n* (%)	3 (3.6)	2 (5.4)	1 (2.2)	0.847
Myalgia/arthralgia, *n* (%)	13 (15.7)	6 (16.2)	7 (15.2)	1
Cephalea, *n* (%)	35 (42.2)	11 (29.7)	24 (52.2)	0.067
Vomiting, *n* (%)	7 (8.3)	4 (10.5)	3 (6.5)	0.791
Abdominalgia, *n* (%)	7 (8.3)	5 (13.2)	2 (4.3)	0.290
**Disease severity**				
Asymptomatic, *n* (%)	13 (15.5)	4 (10.5)	9 (19.6)	0.143
Minimal, *n* (%)	23 (27.4)	15 (39.5)	8 (17.4)	
Mild, *n* (%)	43 (51.2)	17 (44.7)	26 (56.5)	
Moderate, *n* (%)	5 (6)	2 (5.3)	3 (6.5)	
**SARS-CoV-2 Variant**				
Others, *n* (%)	16 (19)	15 (39.5)	1 (2.2)	**<0.001 ****
Omicron, *n* (%)	68 (81)	23 (60.5)	45 (97.8)	

Clinical course of COVID-19 with respect to vaccine status (only first infections considered). IQR = interquartile range. * Hemoptysis occurred already before COVID-19 pandemic. ** Initially, no vaccinations were available. *** Younger patients were ineligible for vaccination due to age criteria.

**Table A2:** Uni- and multivariable models for the time to first infection.

		Univariable				Multivariable			
Variable	Comparison	HR	Lower CL	Upper CL	*p*-Value	HR	Lower CL	Upper CL	*p*-Value
Age at pandemic start (years)		1.008	0.964	1.055	0.721	1.019	0.972	1.068	0.432
BMI Z score		1.326	1.059	1.661	0.014	1.379	1.09	1.745	0.007
CFTR mutation type	F508del heterozygous vs. F508del homozygous	0.843	0.512	1.389	0.502				
	F508del homozygous vs. other	1.044	0.576	1.892	0.886				
Sex	Male vs. female	1.429	0.91	2.243	0.121				
Pancreatic insufficiency	Yes vs. no	0.764	0.427	1.368	0.365				
CFRD	Yes vs. no	0.9	0.363	2.231	0.82				
CFTR modulator	Yes vs. no	0.972	0.565	1.671	0.918				
CFTR modulator type	HEMT vs. no	1.135	0.356	3.621	0.831				
	No HEMT vs. no	0.942	0.524	1.694	0.842				
FEV1% *		1.012	0.994	1.03	0.18				
FEV1 Z score *		1.16	0.939	1.433	0.169				
Lowest HbA1c *		0.732	0.383	1.397	0.344				
Lowest LCI *		0.952	0.839	1.08	0.443				
*Pseudomonas aeruginosa*	Yes vs. no	0.889	0.511	1.545	0.676				
*Achromobacter xylosoxidans*	Yes vs. no	0.805	0.325	1.997	0.64				
*Serratia marcescens*	Yes vs. no	0.328	0.046	2.358	0.268				
*Burkholderia cepacia*	Yes vs. no	0.64	0.088	4.661	0.66				
*Aspergillus fumigatus*	Yes vs. no	0.581	0.267	1.267	0.172				
*Staphylococcus aureus*	Yes vs. no	0.585	0.29	1.18	0.134				
*Haemophilus influenzae*	Yes vs. no	1.862	1.18	2.94	0.008	2.061	1.291	3.292	0.002

BMI = body mass index, CF = cystic fibrosis, CFRD = CF-related diabetes, CFTR = cystic fibrosis transmembrane conductance regulator, CI = confidence interval, CL = confidence level, FEV1 = predicted forced expiratory volume in 1 s [28], HEMT = highly effective modulator treatment, HR = hazard ratio, LCI = lung clearance index, OR = odds ratio. * pre-pandemic best value between 31 January 2019, and 1 February 2020. Airway colonization according to Leeds criteria 12 months before beginning of pandemic.
